# Five Lessons Learned From Randomized Controlled Trials on Mobile Health Interventions: Consensus Procedure on Practical Recommendations for Sustainable Research

**DOI:** 10.2196/20630

**Published:** 2021-02-08

**Authors:** Daniel Pach, Alizé A Rogge, Jiani Wang, Claudia M Witt

**Affiliations:** 1 Institute for Social Medicine, Epidemiology and Health Economics Charité – Universitätsmedizin Berlin, corporate member of Freie Universität Berlin, Humboldt-Universität zu Berlin, and Berlin Institute of Health Berlin Germany; 2 Institute for Complementary and Integrative Medicine University of Zurich and University Hospital Zurich Zurich Switzerland; 3 Department of Psychosomatic Medicine, Center for Internal Medicine and Dermatology Charité – Universitätsmedizin Berlin, corporate member of Freie Universität Berlin, Humboldt-Universität zu Berlin, and Berlin Institute of Health Berlin Germany; 4 Center for Integrative Medicine University of Maryland School of Medicine Baltimore, MD United States

**Keywords:** mHealth, mobile apps, pain, behavior change techniques (BCTs), recommendations

## Abstract

**Background:**

Clinical research on mobile health (mHealth) interventions is too slow in comparison to the rapid speed of technological advances, thereby impeding sustainable research and evidence-based implementation of mHealth interventions.

**Objective:**

We aimed to establish practical lessons from the experience of our working group, which might accelerate the development of future mHealth interventions and their evaluation by randomized controlled trials (RCTs).

**Methods:**

This paper is based on group and expert discussions, and focuses on the researchers’ perspectives after four RCTs on mHealth interventions for chronic pain.

**Results:**

The following five lessons are presented, which are based on practical application, increase of speed, and sustainability: (1) explore stakeholder opinions, (2) develop the mHealth app and trial simultaneously, (3) minimize complexity, (4) manage necessary resources, and (5) apply behavior change techniques.

**Conclusions:**

The five lessons developed may lead toward an agile research environment. Agility might be the key factor in the development and research process of a potentially sustainable and evidence-based mHealth intervention.

## Introduction

Forecasts suggest that digital health might disrupt health care [1]. However, the increasing importance of mHealth technology such as tracing apps against COVID-19 has highlighted that such disruption is already happening. In Germany, the new Law for Digital Health Applications (Digitale-Versorgung-Gesetz [DVG]) [2] might provide further support for this trend. The DVG aims at achieving better coverage of patients through digitization and innovation by implementing the entitlement of insured individuals to digital health apps and allowing physicians to prescribe apps such as mobile Health (mHealth) interventions, which are then reimbursed as medical interventions by the statutory health insurances. The DVG also aims to expand the telematics structure in health care, entitle patients to digital apps, promote the development of digital innovations, and hence financially support developers in the field in cooperation with the statutory health insurance companies [2,3]. Given these new opportunities for the integration of mHealth interventions in Germany, the quality of their development is of major importance.

In digital apps, continuous therapy support and finely graduated feedback on user behavior have great potential not only to affect the health professional-patient relationship but also to change the mode of treatment [4] and prevention in general. Modern medical treatments are usually based on an evidence-based medicine (EBM) approach integrating individual clinical expertise, best external evidence, and patient preferences [5]. Some mHealth app interventions have been able to apply the EBM structure [6], and evaluation frameworks [7] for these interventions have been developed. However, to date, it seems that mHealth rarely follows this EBM principle [3,8]. The main reason for this lack might be that established research methods cannot follow the pace of technological advances and accompanying digital business models. Although the development of a new drug might take 10 to 15 years, a new iPhone, including its new operating system, is released every year, and an app can even be developed within a few months, or even weeks when resources are unlimited. App development for commercial information technology projects is usually realized by professional multidisciplinary teams often lacking clinical trial expertise. These competencies exist in academic and commercial clinical research. In turn, these organizations might greatly benefit from knowledge of the commercial technology sector, including expertise in design, product, or business development. According to a review of mindfulness apps [9], 606 iPhone apps could be identified, but only 23 of these apps actually provided mindfulness training, and only 1 app was supported by a randomized controlled trial (RCT) [10]. Thus, to develop a sustainable evidence-based mHealth app, we consider it necessary to bring these different perspectives closer together.

Ben-Zeev et al [11] shared their experiences and strategies of developing mobile interventions in a review in 2015, mentioning some mHealth challenges such as evolving technology, mobile phone selection, mHealth system “bugs and glitches,” and others. However, technological advances in the last 5 years have shifted mHealth research strategies away from technical concerns toward sustainability and multidisciplinary integration.

In this paper, we provide five recommendations for future app developers based on our expertise gained from four mHealth RCTs for the pain conditions dysmenorrhea [12-14], and back pain and neck pain [15] in a clear concise way. These lessons might accelerate the development of mHealth interventions and their evaluation by RCTs.

## Methods

### Approach and Aims

We are a German research team with expertise in integrative medicine and cognitive behavioral therapy consisting of two medical doctors, one public health researcher, and one behavioral cognitive psychologist. Moreover, the team is experienced in nondigital as well as digital clinical research using RCTs, mixed-methods approaches, and stakeholder engagement. Since 2012, we have developed and evaluated four app-based mHealth interventions for the pain conditions dysmenorrhea [12-14], and back pain and neck pain [15].

This paper aims to support developers of mHealth interventions in the early stages of their career. Toward this end, we applied a continuous consensus procedure. Content units were identified by discussions in multiple rounds to cluster the selected topics using inductive and deductive coding strategies, as well as mind mapping. Topics were categorized by two reviewers (AR and DP) and any discrepancies were resolved in face-to-face discussions.

The lessons described in this paper are based on our experience conducting the four app-based mHealth RCTs described in the following section.

### App-Based mHealth RCTs

#### Luna.

The evidence-based app Luna. [14] (pronounced “Luna period”; trial registration: ClinicalTrials.gov NCT03432611) aims to reduce menstrual pain in women with primary dysmenorrhea (aged 18 to 34 years) by providing different self-care strategies such as self-applied acupressure, exercise, and yoga. In this randomized pragmatic trial, one group of women had access to a full-featured study app consisting of a combination of acupressure and additional self-care features, while the two control groups had either access to acupressure or self-care features only. The primary outcome of the study was the mean pain intensity measured with the in-app numerical rating scale (NRS), ranging from 0 (no pain) to 10 (most intense pain imaginable), on the painful days during the sixth menstruation after starting the intervention. Follow-up measures were collected during 12 menstruation cycles. The app was developed together with an external agency for the iOS platform using the Apple ResearchKit framework. We aimed to include 594 participants in the study.

#### AKUD

The app-based mHealth intervention AKUD [13] was the precursor study to Luna. [14], which aimed to investigate the effectiveness of acupressure. In this two-armed randomized pragmatic trial, 221 women aged 18 to 34 years with cramping pain during their menstruation were included and randomized either to the acupressure or usual care group. The primary outcome was the mean pain intensity measured with the in-app NRS, ranging from 0 (no pain) to 10 (most intense pain imaginable), on the painful days during the third menstruation. This app was developed together with an external agency for the iOS and Android platform.

#### RelaxBack and RelaxNeck

RelaxBack (trial registration: ClinicalTrials.gov NCT02019498; https://www.clinicaltrials.gov /ct2/show/NCT02019498) for chronic back pain and RelaxNeck (trial registration: ClinicalTrials.gov NCT02019134; https://www.clinicaltrials.gov/ct2/show/NCT02019134) for chronic neck pain [15] were evaluated in separate randomized pragmatic trials with the same research design. In both trials, the intervention group received digital instructions on three relaxation techniques as self-care strategies aiming to reduce pain, whereas the control group only documented their symptoms. In total, 270 participants (aged 18 to 65 years) were included in the trials. As the primary outcome, the mean pain intensity during the first 3 months of app usage was measured. These apps were based on the source code of the app AKUD with design changes according to the intervention needs.

## Results

### Lesson 1: Explore Stakeholder Opinions

Research is often conducted from the perspectives of health professionals or researchers, and other perspectives such as those of the patients, customers, and policymakers are neglected. As described in previous research, the sustainability of a developed mHealth intervention often meets obstacles when not in line with the interests of the ministries of health (eg, national strategic goals and laws), especially when it comes to data security; hence, the app developed might not be appropriate for the present infrastructure [16]. Moreover, most mHealth interventions in the form of RCTs are developed as single-stage interventions; however, popular apps usually combine multiple-stage interventions. Hence, the sustainable use of a well-developed app might not only be dependent on its effectiveness but also on its multipurpose applicability to the target group [17]. Accordingly, neglecting the opinions of stakeholders can lead to trials that do not meet the needs of their target group, evaluate irrelevant outcomes, and are therefore not sustainable. Moreover, the user’s app experience is greatly influenced by factors such as design, language, and adaptivity; in turn, the effects of these factors might broadly be influenced by user characteristics such as age, gender, and education. Therefore, we recommend involving stakeholders to understand the target group as early as possible for ensuring a sustainable app and trial.

Example: We involved stakeholders (college students, affected young women, a teacher, a gynecologist, and researchers) in the preparation of our AKUD trial on menstrual pain. In focus group discussions and interviews, stakeholders (potential trial participants) argued against the originally planned relaxation component as part of the intervention due to its length, which resulted in excluding this part from the trial. Moreover, the stakeholders favored an increase of the frequency of questions at the cost of the number of items in each questionnaire.

### Lesson 2: Develop the mHealth App and Trial Simultaneously

To show efficacy, effectiveness, and cost-effectiveness [18,19] of an mHealth intervention, a clinical trial is required. An app and trial each come with their own specific characteristics and needs. An app may be determined by the platform it is available for, its design language, its respective features, or its possible dependencies on valid sensor data or a server. These aspects of the app directly affect the complexity (eg, study sample and adherence) and privacy aspects (eg, data sharing) of the corresponding trial, thereby affecting the necessary resources and development time. Moreover, the choice of hardware and software may influence the structure of the trial, since not every feature is available on a given operating system or device, which may significantly shape the scale of the intervention [11]. On the other side, a trial may be determined by the target group, the clearly defined intervention and control settings, and its research outcomes. These aspects of the trial directly affect the choice of app platform (eg, platform preferences of target populations have to be considered), design of the app (eg, use of age-specific graphical elements), included features (eg, questionnaires), and their technical realization (eg, use of the smartphone’s camera). Previous research has shown that approximately half of study results in mHealth interventions are either unclear or negative; however, the number of mHealth interventions and their popularity are consistently increasing [17,20]. This clearly shows a gap necessary to explore in this field of research, and emphasizes the urgent need for well-planned and performed RCTs that are flexible to the field of mHealth interventions.

Therefore, we recommend that an mHealth app always be developed simultaneously with its respective trial to avoid a waste of time and resources.

Example: During development of the Luna. app [14], we decided to use the data collected for outcome measurement, the NRS for pain ranging from 0 (no pain) to 10 (most intense pain imaginable), and number of activities against period pain as an additional feature for the app dashboard. In this way, the study participants could directly benefit from the data collected for the trial. Moreover, sharing these data with the study participants also impacted the app intervention itself, as this new feedback feature could be considered as a behavior change technique (BCT) [21]. With this approach, we not only added a new feature to the app but we also might have even improved the participants’ study adherence.

### Lesson 3: Minimize Complexity

Currently, mHealth apps need to be developed by multiprofessional teams with expertise in areas such as iOS and Android development, design, backend development, regulatory affairs, psychology, and business development; hence, complexity is already high. Considering the amount of time, expertise, and resources needed for a clinical trial, the appropriate clinical evaluation of mHealth apps substantially adds a magnitude of complexity. Many factors of app and trial development cannot be neglected because of increased user expectations as well as technological and regulatory standards. Moreover, it is difficult to ensure a focused analysis of the interventional effect because mHealth apps are complex interventions including features such as BCTs, combinations of different therapeutic approaches (eg, exercise and diet), and connected devices and services, which are prone to regulation within the health care sector. Knowing that three-quarters of mobile phones are being used in low- or middle-income countries, minimizing complexity might also be beneficial for the sustainability of the mHealth interventions developed, since the latest versions of devices or repair services might not always be within the user’s reach, especially in rural areas [22]. The market of mHealth interventions is often trend-based and dependent on current preferences of the target group. An evidence-based mHealth intervention must therefore not only focus on the quality of the interventions provided but also be able to react timely to changes in the market (eg, software or hardware update) so as to maintain interest of the target group or avoid user frustration. In addition, with a less complex app, the development team might be able to react to bugs or market changes more quickly. To increase the speed of development and the availability of the mHealth intervention for users, we recommend minimizing complexity.

Example: To reach a broad target group, the app AKUD was developed for the two platforms Android and iOS with consideration of their differences in user interface design, general design language, and technological base. However, this approach made it more complex to standardize the intervention, and to develop, test, and support the app, while only gaining a potentially more diverse target group.

### Lesson 4: Manage Necessary Resources

The multidisciplinary team necessary for mHealth app development is usually not available in a research setting. Resources such as designers, frontend or backend developers, and access to technical infrastructure need to be taken into account in addition to the research resources. A lack or insufficient management of these resources leads to a longer time in development, potentially higher financial burden, and less substantial results. We therefore recommend performing prior analyses of existing and necessary resources for app and trial development to adequately manage the mHealth study necessities, and to increase the overall speed of app development to keep up with the rapidly progressing market.

Example: The development of our Luna. app [14] was based on previous app and trial experiences of the AKUD trial. During the preparation of the new trial, the effort for ethical approvals in the participating countries, requiring staff and time, was underestimated. Therefore, the development of the app and the study start were substantially delayed, and funding of research staff became more difficult.

### Lesson 5: Apply BCTs

Besides scalability and efficiency, behavior change is a key component of mHealth [21]. Michie et al [23] defined the smallest, observable, replicable intervention component with the potential to bring about change in behavior as a BCT [14,24]. BCTs could also be defined as “a systematic procedure included as an active component of an intervention designed to change behavior” [23]. In digital interventions, app features and functions could be designed based on different BCTs to improve user engagement [25]. For example, “prompts/cues” could be implemented as an app notification to remind users to fill in questionnaires. Feedback on behavior could potentially maintain users’ motivation by providing instant feedback. Further, “goal-setting” and “self-monitoring” are also commonly implemented BCTs in smartphone apps. In a systematic review, 344 BCT apps were reviewed and rated [26]; however, on average, these apps only showed low to moderate functionality, meaning that only a slight amount of BCT was used, and therefore the full spectrum of potential behavior change due to BCTs was not unfolded.

We recommend involving behavior change specialists and to perform early user testing. As a fundament, the BCT taxonomy [21] and the behavior change wheel framework [27] might be helpful.

Example: The development of our RelaxNeck and RelaxBack studies was based on previous app and trial experiences of the AKUD trial. Although we also included stakeholders in their preparation, we did not involve specialists for BCTs. Therefore, user interaction was neither based on theory nor on defined BCTs. This lack of expertise might have impacted the effectiveness of the app and the results of the respective trial. We had learned from this experience and validated the application of the BCTs implemented in the Luna. app [14] by involving two independent raters with BCT expertise who had experienced the finalized full-featured app but who had not been part of the app development process.

## Discussion

In this project, we have developed five lessons from the practical experience we gained in developing four mHealth interventions and evaluating them with RCTs. Using inductive and deductive coding strategies in this consensus procedure, we developed the following lessons: (1) explore stakeholder opinions, (2) develop the mHealth app and trial simultaneously, (3) minimize complexity, (4) manage necessary resources, and (5) apply BCTs. These lessons might be useful for researchers, entrepreneurs, or other groups dealing with mHealth interventions in an early stage, and might support faster access to evidence-based mHealth interventions that are more sustainable.

We are aware that we cannot cover all aspects of app and trial development for mHealth interventions. The lessons are derived from only four studies of our research group and numerous discussions with startups. Therefore, the applicability of these lessons might be limited due to the focus on one research group, their experiences in Germany only, and the involved professions (medical doctors, psychologists, public health specialist, clinical researchers). These lessons do not cover the important topics of funding, necessary professional qualifications, as well as regulations such as the European Union General Data Protection Regulation and the European Medical Device Regulation. However, these aspects very much depend on individual settings, and therefore general lessons should not be defined. Moreover, the recommendations we made in this project were not tested in a prospective clinical trial; therefore, we cannot make assumptions about the effects (eg, explanation of variance) of each of the recommendations. To conclude about the effectiveness of each lesson (eg, for app engagement), two-armed trials with head-to-head comparisons might be necessary.

Based on nearly 10 years of experience with the applications of RCTs for mHealth interventions, all lessons were derived from actual hands-on experience and were later condensed to allow easy access for researchers and entrepreneurs new to the field. In the future, we consider that an implementation science approach would be helpful to actually measure aspects such as sustainability or the importance of a fast development process in mHealth trials. In public health, the Re-Aim framework aims to improve sustainability and implementation of behavioral interventions by focusing on five aspects: reach, effectiveness, adoption, implementation, and maintenance. Transferring this framework to the field of mHealth interventions might also enhance sustainable development and the overall quality of trials. We hope to contribute new aspects in addition to existing guidance documents of other research groups raising concerns to the current development processes in the field of mHealth [19-21,28].

The practical lessons we learned may best unfold in a research environment that uses agile techniques originally borrowed from software development [29], as we believe that agility might be the key factor for the accelerated development of a sustainable evidence-based mHealth intervention.

## Abbreviations

BCT: behavior change technique
DVG: Digitale-Versorgung-Gesetz (Law for Digital Health Applications)
EBM: evidence-based medicine
mHealth: mobile health
NRS: numerical rating scale
RCT: randomized controlled trial
